# Pain Among Latvian Workers: General Prevalence vs. Registered Occupational Diseases

**DOI:** 10.3389/fpubh.2022.844525

**Published:** 2022-04-29

**Authors:** Darja Kaluznaja, Jelena Reste, Ivars Vanadzins, Svetlana Lakisa, Maija Eglite

**Affiliations:** ^1^Department of Occupational and Environmental Medicine, Riga Stradinš University, Riga, Latvia; ^2^Institute of Occupational Safety and Environmental Health, Riga Stradinš University, Riga, Latvia

**Keywords:** disability, employee, musculoskeletal, occupational disease, overload, pain, prevalence

## Abstract

The problem of painful and disabling work-related musculoskeletal disorders (MSDs) is increasing in many employment sectors of Latvia. Official statistics may underestimate the proportion of affected employees, causing delays in preventive interventions, ineffective rehabilitation, and a reduction of the workforce. This study investigated the prevalence of painful health conditions among Latvian workers by analyzing survey data and comparing these to official statistics on registered occupational diseases (ODs). A total of 2,446 workers participated in the state-level “Work conditions and risks in Latvia, 2017–2018” survey that included questions about pain lasting longer than 3 days during the previous year. The frequency of subjective reports on the presence, severity, and location of pain and related behaviors was assessed in relation to sex, age, education, and job position. Descriptive statistics and cross tabulation with the chi-squared test as well as multinomial logistic regression were applied to the data. Overall, 27.7% of respondents (*n* = 678) reported experiencing pain, predominantly in the lower back (14.3%) and mostly moderate (47.1%) or severe (45.1%). Only one-fifth of respondents (20.5%) took sick leave from work and one-third (29.0%) did not take action to alleviate their pain. Participants aged 55–74 years had a significantly higher odds ratio (OR) for pain in at least one body region—including hands and legs—than those aged 18–24 years. Women had significantly higher odds of headache (OR = 2.55) and neck pain (OR = 1.85) than men. Respondents with a primary or elementary education level had higher odds of pain in at least one body region (OR = 1.60) and in the lower back (OR = 1.86), while those with secondary education had higher odds of pain in hands (OR = 1.51) than employees with higher education. Unskilled workers had significantly higher odds of pain in hands (OR = 2.42) and legs (OR = 2.12) than directors. Official data revealed a dramatic increase in the proportion of MSDs and related disabilities in the last decade, reaching 75.5% of all first registered ODs in 2019. These results demonstrate a high prevalence of painful conditions among Latvian employees; urgent attention to diagnostics, treatment, and prevention is needed to ensure the musculoskeletal health and productivity of this population.

## Introduction

The International Association for the Study of Pain (IASP) defines pain as “an unpleasant sensory and emotional experience associated with, or resembling that associated with, actual or potential tissue damage” (1). Pain is always a subjective feeling but its extent and development is influenced by many biological, social, and psychological factors; it can be categorized as acute, subacute, or chronic according to the duration (2, 3). Chronic pain is defined by IASP as pain that lasts for at least 3 months, which is equal to the amount of time required for the inflammation to subside or an acute condition to heal (4). Epidemiologic studies have reported a prevalence of pain-associated conditions of 10–40%; 10-year rates determined by age–period–cohort modeling have projected an increase in pain prevalence over this period, especially in females and older people (5). In Europe, more than 50% of adults reported experiencing headache in the last year (6). Even minor pain can affect work productivity, increase absenteeism, and in chronic or severe cases, cause disability (7, 8). To understand the full impact of pain on wellbeing, it is important to consider its prevalence, intensity, and the affected individual's levels of emotional stress and dysfunction in daily life activities. Current research focuses mainly on the occupational and socio-economic factors that contribute to the development of overload-related painful conditions and can be in context with employability and disability levels.

Musculoskeletal disorders (MSDs) are widespread, painful, and limit mobility and the performance of everyday activities; this not only impacts individuals' quality of life but also contributes to high disability and absenteeism rates worldwide that impose a considerable economic burden (9–11). Musculoskeletal pain without treatment can lead to more severe disease, resulting in disability and labor shortage. Moreover, employees may return to work only to face the same occupational hazards that caused their disorder, resulting in exacerbation and a chronic medical condition that jeopardizes their future ability to work. Early identification of risk factors for work-related MSDs is important to preserve the health of workers and prevent workforce attrition.

Working conditions in Latvia have improved in the last two decades, especially in terms of workplace safety, accessibility of individual protective equipment provided by employers, new machinery and automatization of processes, as well as air quality at workplaces. However, physical strain, poor ergonomics, and lack of human resources are major problems in many industries. The level of employment in the country is fluctuating, affected by many economic and social factors. High levels of unemployment were present periodically reaching maximal levels during economic crisis, e.g., 21.3% in 2010, but decreasing in periods of high economic activity with a minimal level of 5.3% in 2007 or 6.0% in 2019. While there has been progress in social insurance and worker protection that makes it possible to register and receive state compensation for occupational disabilities and diseases, these can take time to develop and their health effects can persist for a long period. Most cases of occupational disease (OD) in Latvia are registered in late stages when the worker is already disabled, treatment and rehabilitation are ineffective, and return to the labor market is no longer possible. For instance, in 2012, 83.3% of all patients with newly registered ODs were already disabled, and their mean age at the time of registration was 53 years (12). Official statistics on disability, sick leave, and ODs reflect only the most severe cases (i.e., when a person was forced to seek professional medical help), especially as some people continue working despite their pain and it therefore remains undiagnosed (13, 14). Data on the hidden prevalence of painful conditions in the working population is necessary to determine the impact of pain on the state budget (sickness benefits), company costs (e.g., sick leave payments and employee replacement), and expenses related to employees' treatment (15).

The present study investigated the prevalence of painful health conditions among Latvian employees using state-level survey data, which were compared to official statistics on registered ODs and disability in Latvia. The two specific research questions were as follows. (1) Among Latvian workers, which sociodemographic groups have the highest prevalence of pain in specific locations (and therefore need the most support in terms of prevention of painful diseases)? (2) How well do official statistics on ODs represent the extent of painful conditions in the Latvian workforce?

## Materials and Methods

The data analyzed in this study were derived from two sources: (1) survey data from the “Work conditions and risks in Latvia, 2017–2018” study; and (2) official statistics on ODs and disability in Latvia. The first contained information on the prevalence of pain among Latvian employees according to pain location (lower back, neck, hands, legs, headache, and others) and odds ratios (ORs) for different sociodemographic variables (sex, age, education, and job position). This part of the study had a cross-sectional design. The second part of the study was based on official data on annual first registered ODs and provide an overview of incidence trends in ODs in Latvia from 1993 to 2019.

### Survey Design and Population

The “Work conditions and risks in Latvia, 2017–2018” study, which was conducted in collaboration with the State Labor Inspectorate of Latvia (16), was a national periodic workforce survey about working conditions in Latvia. It was the most recent of consecutive studies that have been carried out in Latvia since 2005 at certain time intervals (2005–2007, 2009–2010, and 2012–2013) (12, 17, 18) with the aim of evaluating the state of labor safety systems as a basis for state-level decision-making regarding employment and social policy programs. Questions about the experience of pain (Supplementary Material) were included in the survey for the second time (since the 2012–2013 survey).

The digital survey was conducted in April 2018 and included a representative sample of 2,502 Latvian workers who voluntarily answered questions in a structured computer-assisted personal interview format at their place of residence. Data for unemployed or retired persons were not analyzed in this study. In total, 2,446 of the 2,502 workers completed the survey about the presence of pain. Data on affected body parts, age, sex, and job position were analyzed. The age range of respondents was 18–74 years (mean: 42.78 ± 12.53 years), and the proportion of women and men was 56.0% (*n* = 1370) and 44.0% (*n* = 1076), respectively.

The study sample was selected by quota combined with stratified random sampling. To ensure that the sample was representative of the general working population in Latvia, the results before processing were weighted by region and industry according to the Central Statistical Bureau of Latvia. The sample covered all sectors of the national economy and all regions of Latvia. Pain prevalence results were also recalculated per 100,000 employees to enable comparisons with official statistics on ODs and disability. According to Latvian Central Statistical Bureau data, the number of employees at the end of 2018 was 909,800. Data were calculated proportionally and are shown in Tables 1, 2.

**Table 1:** Prevalence of self-reported pain at different locations lasting longer than 3 days in the last year, by sociodemographic group (percentage/number per 100,000 employees).

	**Low back**	**Neck**	**Hands**	**Legs**	**Headache**
**Gender**					
Men	15.1/6,664	4.0***/1,758	5.8/2,535	5.6/2,453	3.6***/1,594
Women	14.1/7.890	7.2***/4,007	5.5/3,107	7.1/3,966	8.8***/4,906
**Age group (years)**					
18–24	13.8**/940	6.0*/409	3.6***/245	4.2***/286	6.6/450
25–34	11.4**/2,739	3.2*/777	3.1***/736	3.4***/818	6.1/1,472
35–44	12.4**/2,944	6.6*/1,554	4.1***/981	4.5***/1,063	6.7/1,594
45–54	16.6**/3,639	7.6*/1,676	6.5***/1,431	7.3***/1,594	6.3/1,390
55–74	18.3**/4,293	5.7*/1,349	9.6***/2,249	11.3***/2,657	6.8/1,594
**Education**					
Primary or elementary	20.8*/1,431	6.5/450	4.8/327	6.5/450	6.0***/409
Secondary	15.2*/8,545	5.2/2,944	6.6/3,679	6.8/3,802	4.9***/2,739
Higher	12.4*/4,579	6.4/2,371	4.4/1,635	5.9/2,167	9.1***/3,352
**Job position**					
Director	15.1/1,308	7.1/613	4.7***/409	5.2*/450	6.6*/572
Specialist	11.8/3,843	5.6/1,840	3.5***/1,145	6.3*/2,044	9.0*/2,944
Skilled worker	16.2/6,950	5.7/2,453	5.8***/2,494	5.4*/2,330	5.1*/2,167
Unskilled worker	15.9/2,249	4.9/695	10.7***/1,513	10.4*/1,472	5.2*/736
Other	11.9/204	9.5/164	4.8***/82	7.1*/123	4.8*/82

*p < 0.05, **p < 0.01, ***p < 0.001.

**Table 2:** Prevalence of self-reported pain in at least one body part lasting longer than 3 days during the last year, by sociodemographic group.

	**Yes, *n* (%)**	**Yes, *n*/100,000** **employees**	**No, *n* (%)**	**Total**
**Gender**				
Men	284 (26.4)	11,611	792 (73.6)	1,076
Women	394 (28.8)	16,108	976 (71.2)	1,370
Age group***				
18–24	39 (23.4)	1,594	128 (76.6)	167
25–34	129 (21.9)	5,274	459 (78.1)	588
35–44	147 (25.4)	6,010	432 (74.6)	579
45–54	155 (28.9)	6,337	382 (71.1)	537
55–74	208 (36.2)	8,504	367 (63.8)	575
Education*				
Primary or elementary	61 (36.3)	2,494	107 (63.7)	168
Secondary	379 (27.6)	15,495	995 (72.4)	1,374
Higher	238 (26.3)	9,730	666 (73.7)	904
**Job position**				
Director	57 (26.9)	2,330	155 (73.1)	212
Specialist	213 (26.7)	8,708	586 (73.3)	799
Skilled worker	286 (27.3)	11,693	761 (72.7)	1,047
Unskilled worker	110 (31.8)	4,497	236 (68.2)	346
Other	12 (28.6)	491	30 (71.4)	42

*p < 0.05, ***p < 0.001.

### Survey Data Grouping and Transcoding

The dependent variable in the data analysis was pain lasting longer than 3 days during the previous year. All respondents (*n* = 2,446) who answered the question “Have you had pain lasting longer than 3 days in the last year?” were divided into two groups depending on whether they answered “Yes” or “No” (reference group). Respondents were not required to remember the exact duration of pain. Short-term pain (up to 3 days) can be an acute situation with a high probability of resolving successfully, and was therefore not considered in this study. In contrast, longer pain duration (≥3 days) is remarkable enough to be accurately remembered and has a higher probability of chronicity and disability. The questions pertained to pain during the previous year, as the study was focused on pain as an indicator of diminished wellbeing and prognostic factor for disability in working individuals.

The survey included questions about pain location by broad regions of the body (with the possibility of adding a comment about a specific region) and pain severity scored using a 10-point scale, where 10 indicated very strong pain and 1 was very weak pain. For analysis, the pain was categorized as mild (score of 1–4), moderate (score of 5–7), or severe (score of 8–10).

Responses to the question “How did you deal with your pain?” (answered by 672/678 respondents who experienced pain during the last year) were also analyzed.

Dependent variables were analyzed as pain in at least one body part (lower back, neck, hands, legs, headache, other) or pain in a specific body part (low back, neck, hands, legs, headache). There were 64 answers about other specific locations of pain (e.g., toothache, stomach pain, abdominal pain, pain due to an infectious disease); however, because responses were highly heterogeneous, group “other” was not included in the analysis.

Four sociodemographic factors were investigated as independent variables—namely, sex, age, education, and job position. Age groups were 18–24, 25–34, 35–44, 45–54, and 55–74 years. The 55–74 age group combined the 55–64 and 65–74 year age groups because of the small number of respondents in the latter (*n* = 22).

Education level was classified as primary or elementary, secondary, and higher education. Job position (question: “What is your position in your main job?”) categories were director (senior manager of the institution, executive director, commercial director, chairman of the board, middle manager/head of a department); specialist (senior specialist—e.g., doctor, teacher, lawyer, architect, senior accountant; specialist—e.g., nurse, laboratory technician, technician, inspector, rapporteur, assistant); skilled worker (service and sales worker—e.g., secretary, librarian, postal worker, salesperson, customer service specialist, hairdresser, police officer, firefighter; skilled worker and craftsman; operator of equipment and machinery—e.g., builder, mechanic, confectioner, seamstress, carpenter); unskilled worker; and other (open question).

During data grouping and transcoding, responses of “No answer”, “Hard to say”, or “Other” were excluded from the analysis. Because of the small number of such responses, it was assumed that they do not bias data analysis and interpretation.

### Statistical Analysis of Survey Data

The data were analyzed using SPSS Statistics v27 (IBM, Armonk, NY, USA) and Excel v2111 (Microsoft, Redmond, WA, USA) software. Descriptive statistics were used to characterize the frequency of variables in different groups. Cross-tabulation analysis and the chi-squared test were used to determine whether differences between groups were statistically significant. The association between sociodemographic factors and pain was analyzed by multinomial logistic regression and calculated as ORs with 95% confidence intervals (CIs) adjusted for sex, age, education, and job position in order to identify employee groups at risk of experiencing pain in different locations of the body. The group that did not experience pain was used as a reference for the dependent variable; the specific reference values for independent variables (sociodemographic factors), are mentioned in the results (Tables 1–5). ORs were adjusted for sex, age, education level, and job position.

### Analysis of Official Data on ODs and Disability

To compare survey data with official statistics about ODs and disability rates, data from the Latvian State Register of Occupational Disease Patients, State Labour Inspectorate, State Medical Commission for the Assessment of Health Conditions and Working Ability, and national Centre for Disease Prevention and Control were extracted for the period from 1993 to 2020. The number of workers and Latvian inhabitants by year during the study period was obtained from the Latvian Central Statistical Bureau. Data on disability were recalculated per 100,000 of Latvian inhabitants in the corresponding year. The absolute numbers of first registered patients with ODs and the number of ODs registered for the first time in the corresponding year were recalculated per 100,000 of employees in order to enable comparisons with employee survey data. Given that many ODs develop over a long period, it was not possible to select a single representative year for the comparisons. To illustrate the dynamic pattern of official statistical data influenced by many economic and social factors, not only working conditions and employee health itself it was decided to represent data graphically. Maximal and minimal values were discussed in detail. The analyses were mainly focused on painful ODs such as MSDs. The proportion of some OD groups (musculoskeletal, respiratory diseases, carpal tunnel syndrome) relative to the total number of newly registered ODs was calculated to assess trends over time.

## Results

### Analysis of Survey Data

#### General Trends in Pain Among Respondents

Overall, 27.7% of workers (678/2,446) reported pain in at least one body part that lasted at least 3 days in the previous year. As the study sample was representative of the general employed population in Latvia, this amounted to 27,719 cases of pain per 100,000. Lower back pain was reported by 14.3% of respondents, while pain in other locations showed almost equal prevalence (headache [6.5%], leg pain [6.3%], neck [5.7%], and hand [5.6%]) (Table 3). Nearly half of respondents who experienced pain in at least one body area had moderate (47.1%) or severe pain (45.1%), and only a small proportion (6.8%) reported mild pain. These proportions were similar irrespective of the pain location, with the exception of neck pain, which was more frequently moderate (58.9%) with a smaller proportion of respondents reporting severe pain (36.2%) (*p* < 0.05).

**Table 3:** Prevalence of self-reported pain lasting longer than 3 days during the last year according to pain location and severity.

	***n* [% from total**	***n* per 100,000**
	**number of respondents**	**employees**
	**(*n =* 2,446)]**	
Positive answer about presence of any severity or location pain	678 (27.7)	27,719
**Severity of pain**		
Mild pain	46 (1.9)	1,881
Moderate pain	319 (13.0)	13,042
Severe pain	306 (12.5)	12,510
**Pain location**		
Low back	358 (14.3)	14,636
Legs	158 (6.3)	6,460
Neck	142 (5.7)	5,805
Hands	139 (5.6)	5,683
Headache	159 (6.5)	6,500
Other	64 (2.6)	2,617

We analyzed the sociodemographic profile of workers experiencing pain and found that the unadjusted OR of pain in at least 1 body region was significantly higher among employees aged 55–74 years (1.86, 95% CI: 1.25–2.77) than among those aged 18–24 years (Table 4). The difference was significant after adjusting for sex, education, and job position (1.90, 95% CI: 1.28–2.84). There was no difference between sexes in terms of the odds of pain in at least one area of the body. Workers with primary or elementary education had 1.6 times higher unadjusted OR for pain (95% CI: 1.13–2.26) than those with higher education, a trend that persisted after adjusting for sociodemographic variables (1.74, 95% CI: 1.17–2.60). However, there was no difference in pain in at least one body area according to job position (director, specialist, skilled worker, unskilled worker, other) (*p* > 0.05; Table 2).

**Table 4:** Odds of pain in at least one part of the body lasting longer than 3 days during the last year and association with sociodemographic factors.

	**Pain in at least one body region, OR (95% CI)^**a**^, unadjusted**	**Pain in at least one body region, OR (95% CI)^**a**^, adjusted for gender, age, education, job position**
**Gender**		
Women	1.13 (0.94–1.35)	1.12 (0.93–1.35)
Men	1	1
**Age group**		
18–24	1	1
25–34	0.92 (0.61–1.39)	0.97 (0.65–1.47)
35–44	1.12 (0.75–1.67)	1.16 (0.77–1.75)
45–54	1.33 (0.89–2.00)	1.37 (0.91–2.05)
55–74	1.86** (1.25–2.77)	1.90**(1.28–2.84)
**Education**		
Primary or elementary	1.60**(1.13–2.26)	1.74**(1.17–2.60)
Secondary	1.07 (0.88–1.29)	1.04 (0.83–1.30)
Higher	1	1
**Job position**		
Director	1	1
Specialist	0.99 (0.70–1.40)	1.01 (0.72–1.43)
Skilled worker	1.02 (0.73–1.43)	0.94 (0.66–1.34)
Unskilled worker	1.27 (0.87–1.85)	0.98 (0.65–1.49)
Other	1.09 (0.52–2.27)	1.06 (0.50–2.24)

^a^*The reference group for the group that experienced pain was the group of respondents who did not experience pain during the last year*.

**p < 0.01.

*CI, confidence interval; OR, odds ratio. Numbers in bold indicate statistically significant differences*.

The most common types of pain in women were lower back pain and headache, but in men lower back pain and pain in hands and legs were most prevalent (Table 1). The prevalence of neck pain and headache differed significantly between sexes. The prevalence of pain in all locations increased with age with the exception of headache. Headache was most common in workers with higher education, while lower back pain was mostly reported by workers with a primary/elementary education level. With respect to job position, pain in hands and legs was most prevalent in unskilled workers while headaches were most common among specialists.

#### Pain in Specific Body Regions

The most prevalent location of pain was the lower back. Unadjusted odds of lower back pain were 1.86 times higher (95% CI: 1.22–2.84) among employees with primary or elementary education than among those with higher education (Table 5). The odds remained higher after adjustment for sociodemographic variables (OR = 1.96, 95% CI: 1.20–3.18). The highest prevalence was in the 55–74 year age group (4,293 per 100,000 employees), followed by the 45–54 year group (3,639 per 100,000) and the 18–24 year group (*p* < 0.01; Table 1).

**Table 5:** Odds of different pain locations during the last year and association with sociodemographic factors.

	**Low back pain**, **OR, 95% CI**^**a**^		**Neck pain**, **OR, 95% CI**^**a**^		**Hands pain**, **OR, 95% CI**^**a**^		**Legs pain**, **OR, 95% CI**^**a**^		**Headache**, **OR, 95% CI**^**a**^	
	**Unadjusted**	**Adjusted^**b**^**	**Unadjusted**	**Adjusted^**b**^**	**Unadjusted**	**Adjusted^**b**^**	**Unadjusted**	**Adjusted^**b**^**	**Unadjusted**	**Adjusted^**b**^**
**Men**	1	1	1	1	1	1	1	1	1	1
**Women**	0.92, 0.73–1.15	0.95, 0.75–1.20	1.85**, 1.28–2.67	1.81**, 1.24–2.65	0.96, 0.68–1.36	0.89, 0.62–1.28	1.29, 0.93–1.80	1.20, 0.85–1.70	2.55***, 1.76–3.70	2.38***, 1.63–3.47
**18–24**	1	1	1	1	1	1	1	1	1	1
**25–34**	0.81, 0.48–1.34	0.88, 0.53–1.47	0.52, 0.24–1.15	0.51, 0.23–1.13	0.85, 0.33–2.17	0.88, 0.34–2.27	0.81, 0.33–1.94	0.82, 0.34–1.99	0.93, 0.46–1.86	0.79, 0.39–1.62
**35–44**	0.89, 0.54–1.47	0.96, 0.58–1.60	1.10, 0.54–2.26	1.04, 0.50–2.15	1.16, 0.47–2.89	1.22, 0.49–3.06	1.08, 0.46–2.52	1.09, 0.46–2.56	1.02, 0.51–2.05	0.85, 0.42–1.71
**45–54**	1.24, 0.76–2.04	1.34, 0.81–2.20	1.30, 0.63–2.65	1.20, 0.59–2.47	1.87, 0.77–4.52	1.90, 0.78–4.62	1.79, 0.79–4.08	1.77, 0.77–4.04	0.96, 0.48–1.94	0.80, 0.39–1.63
**55–74**	1.40, 0.86–2.28	1.50, 0.92–2.46	0.96, 0.46–1.98	0.88, 0.42–1.84	2.84*, 1.20–6.71	2.78*, 1.17–6.61	2.91**, 1.31–6.48	2.83*, 1.27–6.32	1.03, 0.52–2.06	0.87, 0.43–1.75
**Primary or elementary**	1.86**, 1.22–2.84	1.96**, 1.20–3.18	1.02, 0.53–1.99	1.33, 0.62–2.85	1.08, 0.50–2.35	0.68, 0.29–1.59	1.13, 0.58–2.20	1.04, 0.49–2.23	0.63, 0.32–1.25	0.97, 0.45–2.08
**Secondary**	1.27, 0.99–1.62	1.24, 0.93–1.64	0.81, 0.57–1.15	0.90, 0.59–1.36	1.51*, 1.03–2.22	1.01, 0.65–1.57	1.17, 0.82–1.65	0.97, 0.65–1.46	0.51, 0.37–0.72	0.65, 0.44–0.96
**Higher**	1	1	1	1	1	1	1	1	1	1
**Director**	1	1	1	1	1	1	1	1	1	1
**Specialist**	0.75, 0.49–1.16	0.77, 0.50–1.20	0.78, 0.43–1.44	0.74, 0.40–1.36	0.73, 0.35–1.54	0.79, 0.38–1.70	1.22, 0.62–2.39	1.28, 0.65–2.52	1.40, 0.77–2.54	1.28, 0.70–2.33
**Skilled worker**	1.09, 0.72–1.64	0.96, 0.62–1.49	0.80, 0.44–1.44	0.83, 0.44–1.55	1.25, 0.63–2.48	1.49, 0.72–3.09	1.05, 0.54–2.04	1.09, 0.54–2.20	0.75, 0.41–1.39	0.88, 0.46–1.63
**Unskilled worker**	1.06, 0.66–1.71	0.81, 0.48–1.37	0.68, 0.33–1.39	0.60, 0.27–1.32	2.42*, 1.18–4.97	2.60*, 1.17–5.77	2.12*, 1.06–4.27	1.76, 0.81–3.78	0.78, 0.38–1.60	0.85, 0.39–1.88
**Other**	0.76, 0.28–2.08	0.70, 0.25–1.95	1.38, 0.44–4.39	1.48, 0.45–4.79	1.01, 0.21–4.79	1.25, 0.26–6.02	1.41, 0.38–5.27	1.57, 0.41–6.01	0.71, 0.16–3.23	0.80, 0.17–3.72

*^a^The reference was employees who did not experience pain during the last year*.

*^b^Adjusted for sex, age, education, and job position*.

*p < 0.05, **p < 0.01, ***p < 0.001.

*CI, confidence interval; OR, odds ratio. Numbers in bold indicate statistically significant differences*.

Slightly lower prevalence was observed for neck pain. Odds of neck pain were ~2 times higher among women than men (OR = 1.85, 95% CI: 1.28–2.67); the highest prevalence of neck pain was observed in the 45–54 year age group (7.6% or 1,676 per 100,000 employees), followed by the 35–44 year group (6.6% or 1,554 per 100,000) and 55–74 year group (5.7% or 1,349 per 100,000). Conversely, age between 25 and 34 years had a protective effect against neck pain compared to the youngest age group (18–24 years), although the difference was not statistically significant.

Odds of hand pain were higher in the 55–74 year age group than in the 18–24 year group (unadjusted OR = 2.84, adjusted OR = 2.78), in unskilled workers vs. directors (unadjusted OR = 2.42, adjusted OR = 2.60), and workers with secondary education vs. those with higher education (unadjusted OR = 1.51; Table 5). However, the difference according to education level was no longer significant after adjusting for sex, age, and job position. Additionally, after adjustment, workers with primary or elementary education had the same odds of pain in hands as those with higher education, although the difference was not statistically significant. Like lower back pain, approximately equal proportions of men and women had pain in hands (Table 1).

Odds of leg pain were 2.91 times higher (95% CI: 1.31–6.48) among employees in the 55–74 year age group than in the youngest age group (18–24 years), and remained higher after adjusting for sex, age, and education level (OR = 2.83, 95% CI: 1.27–6.32). Unskilled workers had 2.12 times higher odds (95% CI: 1.06–4.27) of pain in legs than directors, but the difference was nonsignificant after adjustment. Women had significantly higher odds of headache than men (unadjusted OR = 2.55, adjusted OR = 2.38; Table 5). There were no significant differences across groups according to other sociodemographic factors. Headaches tended to be more frequent among workers with higher education (9.1%) compared to those with a primary or elementary (6.0%) and secondary (4.9%) education level (*p* < 0.001, Table 1). Headache was most prevalent among specialists (9.0%).

#### Workers' Behavior With Respect to Pain

The behavior of workers due to pain varied: most (57.6%, 387/672) visited a doctor but others (29.0%, 195/672) did not take any action, and only one-fifth (20.5%, 138/672) used their sick leave. Other actions by a small number of respondents (<10%) included a change in working method or technique (7.0%, 47/672) or in the pace of work (5.7%, 38/672). A small number of employees (≤ 3.0%) noted that they requested to be sent for a compulsory health examination (3.0%, 20/672), changed jobs (2.4%, 16/672), reached an agreement with their employer to reduce working hours (1.6%, 11/672), or worked from home (1.6%, 11/672). There was no significant difference between women and men in terms of behavior related to pain.

### Analysis of Official Statistics on ODs

#### General Trends

To evaluate the consequences of persistent pain among Latvian workers, we analyzed official statistics on disability and ODs. Because these were not directly comparable to the survey data, only an overview of official statistics is presented after recalculating per 100,000 employees (to make possible the comparison with survey data seen in Tables 1–5). We focused on MSDs, which are among the most painful and widespread work-related conditions.

**Figure 1:**
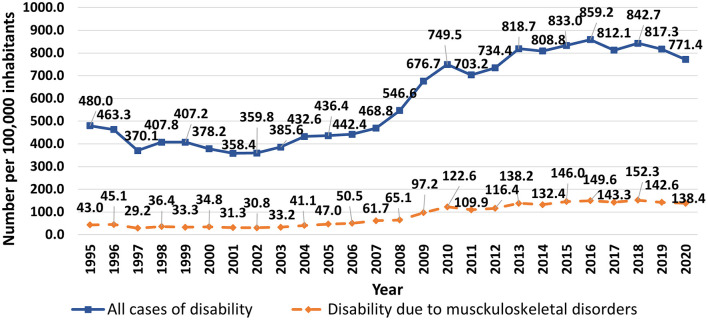
Trends in first registered general disability in Latvia, 1995–2020 (total number of cases and cases caused by musculoskeletal diseases per 100,000 inhabitants).

Disability among general population in Latvia has increased in recent decades (Figure 1). The increases were mainly in oncologic and cardiovascular diseases, with MSDs as the third leading cause of general disability. The proportion of people with MSDs was lowest in 1997 (29.2 per 100,000 inhabitants or 7.9% of all disabled persons registered for the first time in that year, 713/9,048) and highest in 2018 (152.3 per 100,000 inhabitants or 18.1% of all disabled persons, 2,946/16,301).

#### First Registered ODs in Latvia

**Figure 2:**
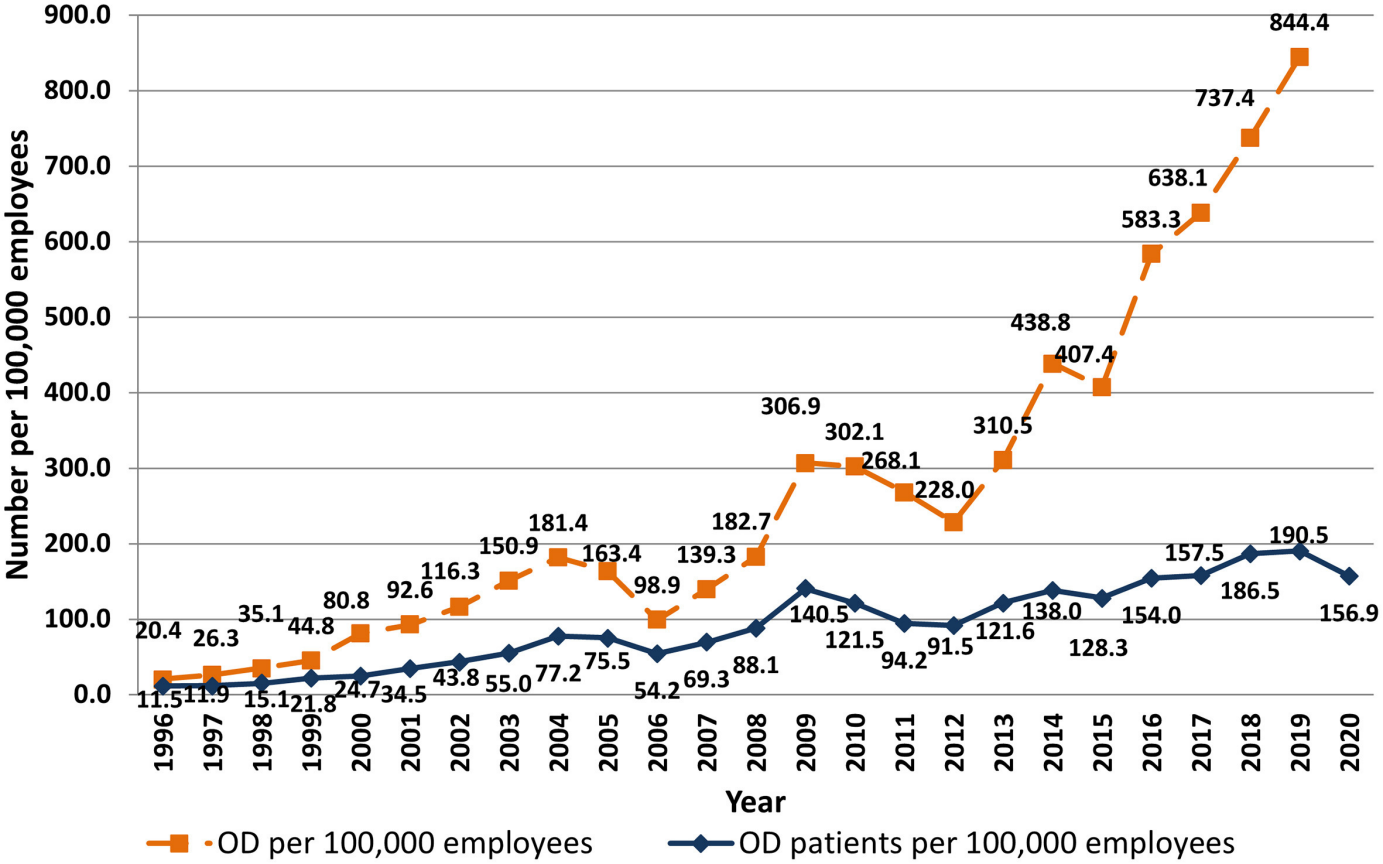
Trends in first registered ODs in Latvia, 1996–2020 (number of patients and number of ODs per 100,000 employees).

ODs are a significant part of previously mentioned statistics on disability. Many people with officially registered OD usually have a registered disability as well. The number of patients with first registered ODs has significantly increased in the last decades, from 11.5 per 100,000 workers in 1996 (*n* = 109) to a maximum of 190.5 per 100,000 (*n* = 1,739) in 2019 (Figure 2). Many individuals registered multiple ODs, such that the number of first registered ODs has increased at a higher rate than the number of patients—e.g., in 1996, there were 20.4 ODs per 100,000 employees (*n* = 194) but in 2019, 844.4 cases per 100,000 (*n* = 7,710).

The proportion of patients with at least one MSD among all officially registered OD cases has also increased over time. In 2018, 97.4% of all newly registered patients with ODs (1,607/1,697) had at least one occupational MSD compared to 30.6% (41/134) in 1995 and 20.3% (24/118) in 1997. Typically, a single individual registered ≥1 MSD; the average number of ODs per person in 2018 was 4.8 in men and 3.7 in women. The mean age of patients with first registered OD in 2018 was 54.3 ± 6.8 years; 87.4% of all newly registered patients were between 45 and 64 years of age, while workers aged 25–34 years and ≥65 years constituted 0.7 and 1.5%, respectively, of this population. The average length of service under hazardous working conditions was 27.0 ± 9.2 years (mostly from 21 to 40 years), implying that many individuals were working for years while they developed ODs. The largest proportion of workers with multiple ODs had painful musculoskeletal conditions related to physical overload. For instance, 98.8% of all patients registered in 2018 had at least 1 OD that developed as a result of exposure to biomechanical and physical stresses.

**Figure 3:**
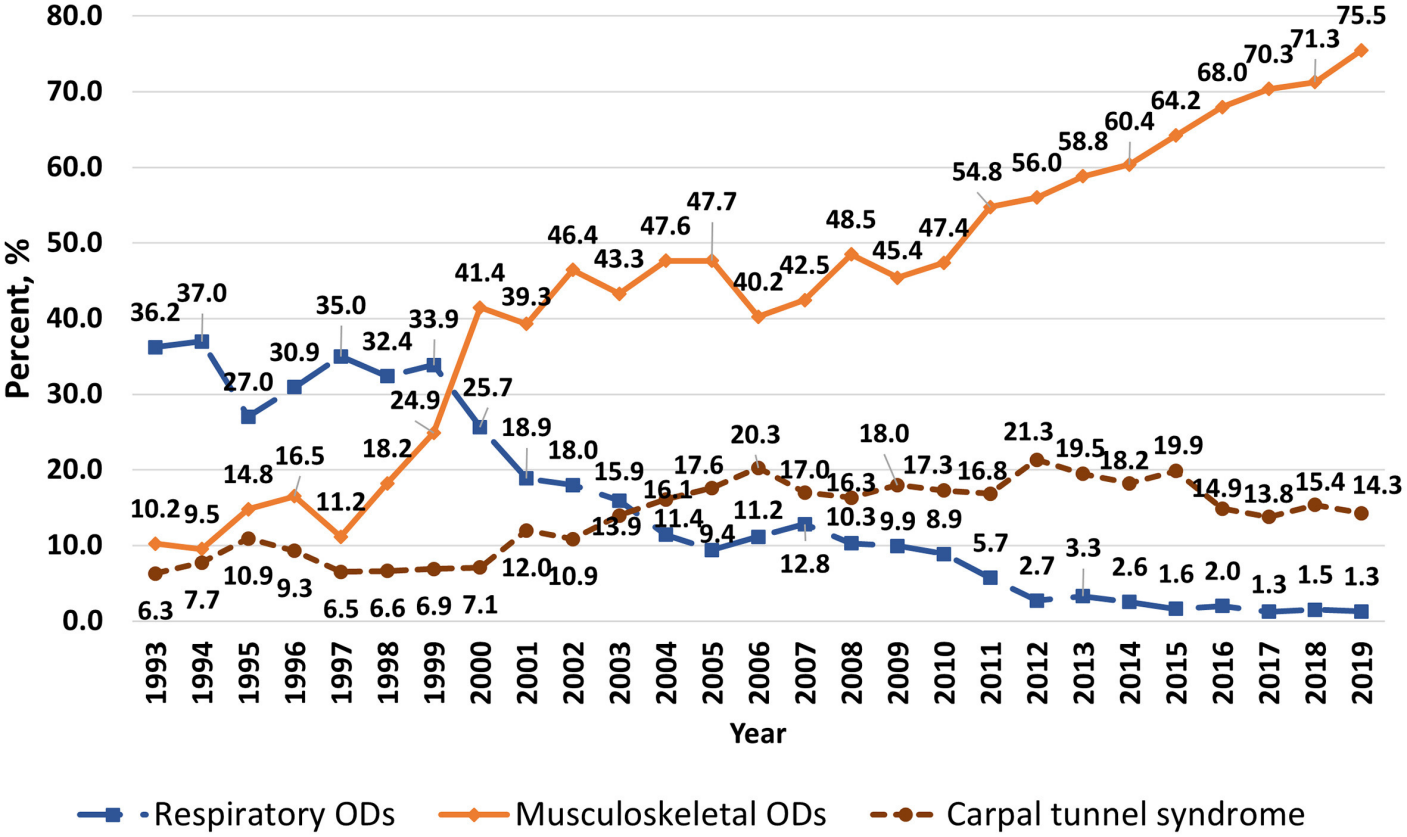
Trends in first registered musculoskeletal and respiratory ODs and occupational carpal tunnel syndrome in Latvia, 1993–2019 (proportion of total number of ODs registered in the year).

The proportion of total first registered ODs that were MSDs has increased in the last decades, especially since 2011, with a concomitant decrease in the proportion of classical ODs such as respiratory diseases (Figure 3). In 1993, respiratory ODs accounted for 36.2% of all first registered ODs and MSDs only 10.2%, but in 2019 the proportions were 1.3 and 75.5%, respectively. The frequency of carpal tunnel syndrome, another painful overload-related disease, has also increased over time, constituting 6.3% of all newly registered ODs in 1993 (*n* = 8), and 21.3% in 2012 (*n* = 425), with even more cases (*n* = 1,100) registered in 2019. However, the relative proportion of first registered ODs that were cases of carpal tunnel syndrome has been decreasing since 2012, reaching 14.3% in 2019.

**Figure 4:**
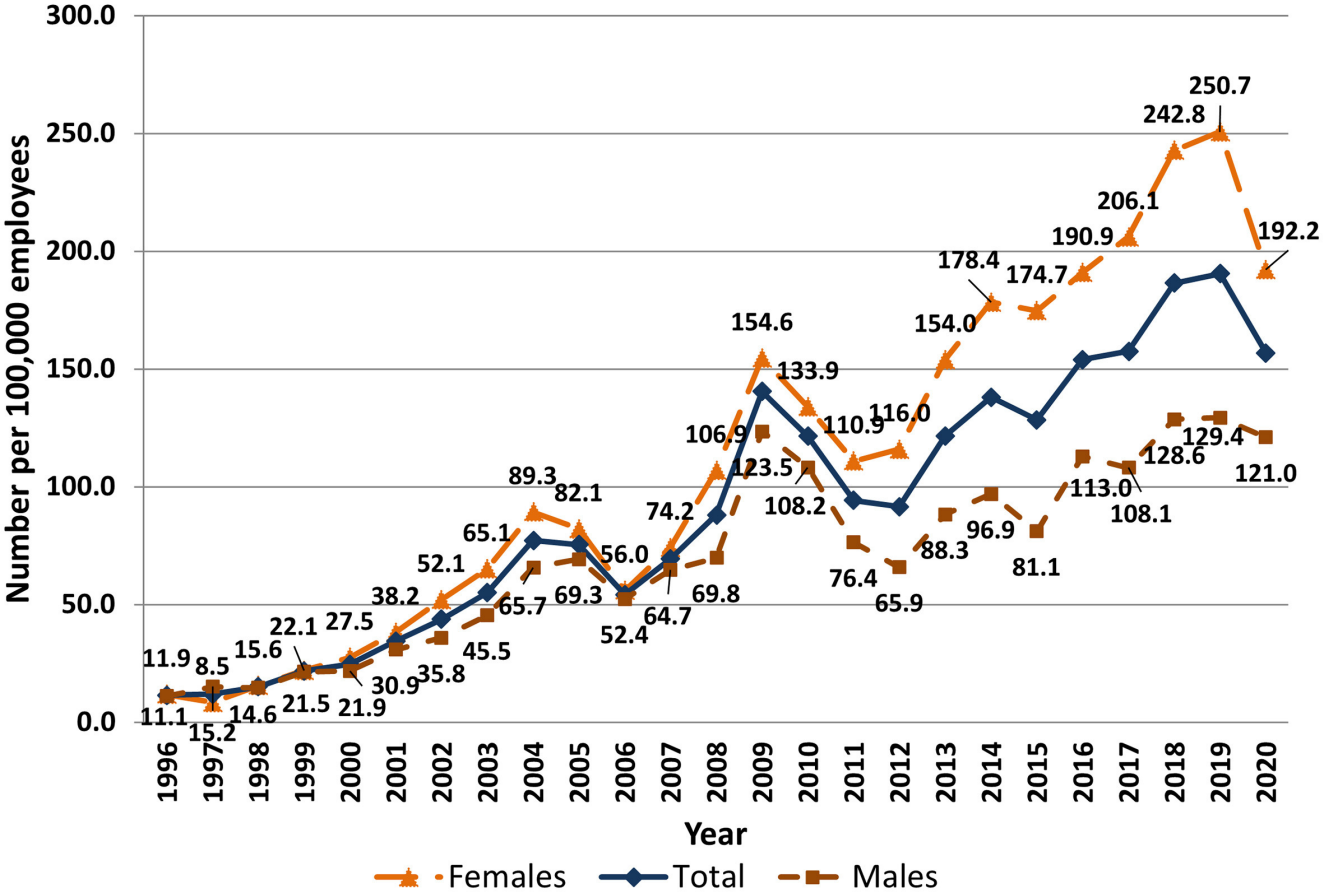
Number of patients with first registered ODs in Latvia by sex and year, 1996–2020 (number of patients per 100,000 employees).

Significant differences in the number of first registered ODs were observed between sexes (Figure 4). For many years more women than men had newly registered ODs, but the disparity has increased in the last decade. For instance, in 2019, double the number of female vs. male patients had first registered ODs (250.7 and 129.4 per 100,000 employed women and men, respectively). The incidence of ODs varied according to job position. In 2018, skilled workers accounted for the largest proportion of OD cases (58.6% or 109.3 per 100,000 employees); this included operators of equipment and machinery (24.4% or 45.5 per 100,000) and skilled workers and craftsmen (6.9% or 31.5 per 100,000). Specialists constituted 17% of newly registered OD cases (32.3 per 100,000 employees), unskilled workers 16.1% (30.1 per 100,000 employees), service and sales workers 16.0% (29.8 per 100,000 employees), and directors 2.7% (4.9 cases per 100,000 employees).

## Discussion

Long-lasting pain can become a chronic condition and disability, but people who are unable to work and are on sick leave may not be included in official statistics on disability and registered ODs. Data on the prevalence of pain in the working population are critical for guiding health and social policy decisions. In this context, the present study analyzed survey data and official statistics on ODs and pain among workers in Latvia. The reasons for pain development can vary and were not considered and only pain lasting longer than 3 days was analyzed in reference to sociodemographic factors. Respondents answered questions about pain during the previous year. Some recall bias was possible, but it was assumed that pain lasting at least 3 days would be accurately remembered. The recalculation of survey and registry-based data per 100,000 employees allowed us to compare the two datasets to obtain a more accurate picture of the prevalence of ODs among Latvian workers. We found that subjectively reported pain was much more common than official statistics would suggest. This was supported by our finding of high disability rates and proportion of individuals with multiple overload-related ODs in the official data. Underestimating the prevalence of painful conditions in workers can undermine the workforce as these disorders can develop into more a debilitating disease. Our findings suggest an ongoing need to improve work conditions in Latvia in order to ensure the health and safety of workers and maximize their productivity.

Physically demanding work can result in the early development and exacerbation of overload-related MSDs. As MSDs are among the most painful, long-lasting, disabling, and widespread health problems in society, these were the main focus of our analysis of first registered occupational MSDs in official statistics. For a long time, precise data on reasons for sick leave taken by employees were not available in Latvia; we therefore compared data on disability and registered ODs to the survey data. To register an OD, the contribution of an occupational hazard to the development of the disorder or condition must be clearly demonstrated, with all other possible reasons excluded; thus, diseases to which non-occupational factors contributed are not registered. Our analysis of official statistics revealed that the number of patients with first registered ODs has significantly increased in recent decades. This may be explained by improvements in legislation pertaining to ODs and workplace accidents and the social insurance system in Latvia, as well as better education of employees about the possibility of registering ODs to receive compensation. Additionally, more cases of painful MSDs have been registered as an OD in recent years. Technologic advances in diagnostic modalities such as ultrasonography, computed tomography, and magnetic resonance imaging have improved the diagnosis of physical overload-related soft tissue damage and other musculoskeletal conditions including lateral and medial epicondylitis, rotator cuff tendonitis, wrist tendonitis and others, allowing workers with musculoskeletal pain to register their condition as an OD.

The analysis of survey data revealed that ~28% or respondents had experienced pain lasting more than 3 days in the previous year; half of these respondents had lower back pain, which was equally prevalent among men and women. Similar results were obtained in a study of chronic musculoskeletal pain among French workers (19), although another study reported that the most common sites of pain were limbs and/or joints (33.3%), back (23.1%), head (11.8%), and neck (8.0%) (15). Neck and shoulder pain have been linked to work-related stress (20) and can predict pain in women (19), but various conditions can contribute to pain; for example, metabolic syndrome increased the occurrence of neck pain, with a stronger association found in men (21). Conversely, positive psychosocial factors can alleviate pain: hand pain was 2.79 times more common in workers who were exposed to uncomfortable tool grips and had little opportunity to sit at work, but the OR was decreased in the context of a favorable psychosocial environment (i.e., satisfaction with the level of work difficulty) (20).

In our study, ODs were more prevalent among women. One possible reason for sex differences is physiologic and anatomic differences that allow men to tolerate greater physical loads than women (22, 23). Other reasons are the larger amount of housework and family care performed by women and the fact that women in Latvia are more health-conscious than men and are therefore more likely to visit a doctor for pain or other reasons. Specifically, the survey revealed higher rates of neck pain and headache among women; this is in line with an earlier findings from a Norwegian study of subjects aged 24–76 years that neck and lower back pain were more prevalent in women than in men (neck: 43.0% vs. 26.9%; lower back: 38.6% vs. 29.3%) (24). Additionally, tension-type headaches were often reported by employees with chronic musculoskeletal pain, and were 2–3 times more common in women than in men (25). Men and women are often exposed to different work environment risk factors (26). A New Zealand study showed that men dominate sectors and occupations involving physically demanding work. However, in a study of self-reported occupational exposures, a high prevalence of weightlifting was observed among nurses (27), which was associated with a significant strain on the lumbar spine (28). Sex differences in experience of pain and ODs should be taken into consideration so that preventive measures can be more effectively targeted (19, 29).

In addition to the observed differences between sexes, there were statistically significant differences in the prevalence of pain across age groups, with older workers (aged 55–74 years) being the most affected and having higher odds of pain in at least one body region, consistent with other reports (30, 31). In a large sample of European adults, the prevalence of self-reported pain was highest in adults aged 45–55 years (23.1%), although these investigators observed the lowest prevalence (5.7%) in the 18–25 year age group (29), in contrast to our results, which showed that workers aged 18–24 years had a higher prevalence of various types of pain (pain in at least one part of the body; pain in lower back, neck, hands, legs; headaches) than the 25–34 year age group. In a previous study conducted in Latvia, most ODs (60.8%) reported by young people were associated with the musculoskeletal and connective tissue systems; the most common risk factors were the lifting of heavy objects, working overtime, and working with computers (12). However, a 14-year prospective study in Norway found that pain prevalence was approximately equal in younger and older respondents, suggesting that the preconditions for pain in later life are established in childhood (32). This is supported by the finding that pain was widespread in many parts of the body in children and adolescents before they entered the workforce (31). Thus, a previous experience of pain can determine pain in later life at all ages (32): a pain experience before the age of 65 years increased the OR for widespread pain including back pain in both women and men (33). Poor and uncomfortable posture at any life stage could lead to back pain, which may be exacerbated by abdominal and back muscle weakness and spinal instability caused by a sedentary lifestyle; moreover, being overweight can overload the spine and impair stabilizing functions (28). In a Canadian study, looking at workers aged 18 to 65 who worked 25 or more hours a week and mostly stood during the working day, the higher odds of having low back pain were among 18–24 years olds (OR = 1.44). In comparison, workers in the predominantly sedentary posture were found to have a significantly higher risk of low back pain in the 40–49 age group (OR = 1.42, 95% CI 0.98–2.05) (34).

Sociodemographic factors other than age also influence the rates of pain among workers. ORs for pain in at least one body region and lower back pain were higher among workers with a primary or elementary education level as compared to other groups, while those with a secondary education had a higher OR for pain in hands. Similar results were found among European adults in the UK, France, Spain, Germany, and Italy, with self-reported pain being higher among adults without a university degree (29). A cross-sectional study of the impact of chronic pain on an individual's employment showed that chronic pain was more prevalent in subjects with primary and secondary education as compared to those with higher education levels (15). We found that job position also affected the rate of self-reported pain: the unadjusted OR for pain in hands and legs was higher among unskilled vs. skilled workers. Unskilled workers are exposed to greater physical demands that can contribute to pain development (35). For instance, a study conducted in New Zealand showed that unskilled workers were over two times more likely than their skilled counterparts (managers and professionals) to lift weights at least 25% of their working time (19). Additionally, MSDs in hands were found to be common among manual laborers (36, 37), while back pain was frequently reported by drivers, handlers of heavy objects, and nurses and other patient-care workers (38–40).

Acquired in the current study prevalence rates and odds ratios for pain together with statistics on occupational diseases are critical work-related indicators that should be considered when planning labor protection policies and organizing health-promoting measures for employees. For instance, special attention should be paid to compulsory health check-ups in identified risk groups to reveal early signs of painful MSDs in employees and start early medical intervention and rehabilitation. Ergonomic and organizational interventions should be planned for those groups of employees who reported higher rates of pain. Health promoting activities should be focused on most severely affected groups, as well as on those who potentially might be impacted by pain later in their life. Current study identified high-risk groups and provided valuable information that can be used for developing new intervention projects in groups of a society where the pain is highly prevalent.

The main strength of the current study was that it provided insight into the prevalence of painful conditions in the working population of Latvia independent of official statistics. Moreover, the comparative analysis of official data on registered ODs and survey results of self-reported pain revealed discrepancies that underscore the value of using multiple independent datasets in population studies. For example, ODs were most frequently officially registered in Latvia by workers aged 45–64 years, but the survey revealed that pain was already present at a much younger age. We also observed differences in pain according to sociodemographic factors—for example, the highest morbidity from ODs was among specialists and skilled workers, whereas pain in hands and legs was most prevalent among unskilled and less educated workers, which is useful information that can guide the implementation of targeted interventions.

However, there were also limitations to this study. First, survey data are subjective and prone to reporting and recall bias. Second, the questions only addressed pain that had emerged in the previous year and had lasted longer than 3 days; no data were available on the number of times respondents had experienced this pain, which could vary from once or multiple times during that year. Third, the definition of pain in various studies differs (according to severity, duration, location, target population group), as studies can be aimed to research pain patterns, mechanisms, diseases, or pain information can be a part of a large national survey. In our study presence of pain was investigated as an indicator of disturbed wellbeing of employees. Fourth, the questions on pain in different parts of the body included a limited number of options for answers (e.g., headache, lower back) but not specific diseases or affected organs, which is why the reasons for pain were not analyzed. Fifth, occupational groups are not homogeneous and comprise employees performing various work tasks, which makes it difficult to measure work-related MSDs in a specific group (36); for example, in the New Zealand study of self-reported occupational exposures, significant differences in factor exposure for a job position was observed across industry sectors (27, 41). Sixth, because of the cross-sectional nature of the survey, it was not possible to draw conclusions on the trends in ODs, for which only official statistics could be used. Finally, our study represents the state of workers in Latvia and cannot necessarily be generalized to the working population in other countries.

## Conclusions

The results of this study indicate that the number of workers in Latvia who are affected by pain may be underestimated by official statistics. The survey results showed that moderate and severe pain lasting at least 3 days was highly prevalent in the Latvian workforce. Lower back pain was most frequently reported, and a higher prevalence of pain was found in older and unskilled workers and employees with a lower education level. Headache and neck pain were more common in women than in men, and the annual number of registered ODs was two times higher in women than in men. Official statistics showed that in the last decade, there has been a marked increase in the number of registered occupational overload-related MSDs and disability due to MSDs, although our findings suggest that these statistics may not fully reflect the prevalence of painful conditions in the working population. As such, there is a need for greater investment in the diagnostics, treatment, and prevention of overload-related MSDs and other painful conditions in Latvia, with a focus on high-risk groups. The prevalence of ODs and risk of pain determined in the present study should be considered when planning labor protection policies and developing health-promoting measures for workers such as compulsory health check-ups in identified risk groups to detect early signs of painful MSDs, which can allow early medical intervention and rehabilitation.

## Data Availability Statement

The raw data supporting the conclusions of this article will be made available by the authors, without undue reservation.

## Ethics Statement

The studies involving human participants were reviewed and approved by the Ethics Committee of Riga Stradinš University. Written informed consent for participation was not required for this study in accordance with the national legislation and the institutional requirements.

## Author Contributions

DK and JR: study conceptualization, data analysis, and manuscript writing. IV: study conceptualization and funding acquisition. SL: assistance with data analysis. ME: study conceptualization, manuscript proofreading, establishment and development of occupational health and safety system in Latvia for >30 years, enabling the gathering of statistics on ODs. All authors participated in manuscript preparation and approved the final version.

## Funding

The study working conditions and risks in Latvia, 2017–2018 was carried out with the financial support of the European Union, European Social Fund, and the Latvian state project improvement of practical implementation and supervision of labor safety regulations (No. 7.3.1.0/16/I/001). Publishing expenses were covered by Riga Stradinš University.

## Conflict of Interest

The authors declare that the research was conducted in the absence of any commercial or financial relationships that could be construed as a potential conflict of interest.

## Publisher's Note

All claims expressed in this article are solely those of the authors and do not necessarily represent those of their affiliated organizations, or those of the publisher, the editors and the reviewers. Any product that may be evaluated in this article, or claim that may be made by its manufacturer, is not guaranteed or endorsed by the publisher.
