# Supplementary material for: Pain Among Latvian Workers: General Prevalence vs. Registered Occupational Diseases
Source: Front Public Health. 2022 Apr 29;10:844525. doi: 10.3389/fpubh.2022.844525 (PMC9099089; doi:10.3389/fpubh.2022.844525)
Supplement: Supplementary file 1 [file Table_1.DOCX]

|  | | | | | | | | | | | | |
| --- | --- | --- | --- | --- | --- | --- | --- | --- | --- | --- | --- | --- |
| SUPPLEMENTARY MATERIAL | | | | | | | | | | | | |
|  | | | | | | | | | | | | |
| Questions were translated from Latvian into English by current research article authors from the official questionnaire.  "Improvement of practical implementation and supervision of occupational safety regulatory enactments" ESF project identification No. 7.3.1.0./16/I/001  **WORKING CONDITIONS AND RISKS IN LATVIA 2017-2018**  **Employee survey ***  **CAPI (computer assisted personal interviews)*, at least 2500 respondents, interview duration not less than in the purchasing study "Working conditions and risks in Latvia 2012-2013" (average interview length 26.1 minutes) | | | | | | | | | | | | |
| QUESTIONS RELATED TO HEALTH DISORDERS AND MANDATORY HEALTH TESTS | | | | | | | | | | | |  |
|  | | | | | | | | | | | |  |
| **B55A. During the last year, have you had pain for more than 3 days?** *Choose one answer.* | | | | | | | | | | | |  |
| 1. | Yes | | | | | | | | | | | ⬜ |
| 2. | No | | | | | | | | | | | ⬜ |
| 98. | Difficult to answer/NA | | | | | | | | | | | ⃝ |
|  |  | | | | | | | | | | |  |
| **B55B. In which location(s) of your body did you have pain?** *Multiple answers are possible.* | | | | | | | | | | | | |
| 1. | In the lower back | | | | | | | | | | | ⬜ |
| 2. | In the neck | | | | | | | | | | | ⬜ |
| 3. | In the hands | | | | | | | | | | | ⬜ |
| 4. | On his legs | | | | | | | | | | | ⬜ |
| 5. | Headaches | | | | | | | | | | | ⬜ |
| 6. | Other location (specify where) __________________ | | | | | | | | | | | ⬜ |
| 98. | Difficult to answer/NA | | | | | | | | | | | ⃝ |
|  |  | | | | | | | | | | |  |
| **B55C. How did you deal with the pain?** *Multiple answers are possible.* | | | | | | | | | | | | |
| 1. | I made a doctor appointment | | | | | | | | | | | ⬜ |
| 2. | I switched jobs | | | | | | | | | | | ⬜ |
| 3. | I changed my working methods or techniques | | | | | | | | | | | ⬜ |
| 4. | I changed the pace of work | | | | | | | | | | | ⬜ |
| 5. | I asked to refer me for a mandatory medical examination | | | | | | | | | | | ⬜ |
| 6. | I used a sick leave | | | | | | | | | | | ⬜ |
| 7. | I agreed with the employer to shorten working hours | | | | | | | | | | | ⬜ |
| 8. | I worked from home | | | | | | | | | | | ⬜ |
| 9. | I didn't do anything | | | | | | | | | | | ⬜ |
|  |  | | | | | | | | | | |  |
| **B55D.**  **Please describe the severity of your pain.**  *Please select the number that best represents your pain intensity on a 0-to-10 scale where 0 = No pain and 10 = Pain of unbearable intensity*. | | | | | | | | | | | | |
| 1. | Very weak | 1 | 2 | 3 | 4 | 5 | 6 | 7 | 8 | 9 | 10 | Very strong |
| 98. | Difficult to answer/NA | | | | | | | | | | | ⃝ |
|  |  | | | | | | | | | | |  |
| **DEMOGRAPHY** | | | | | | | | | | | | |
|  | | | | | | | | | | | |  |
| **R4. Your age:** | | | | | | | | | | | | |
| 1. | Full years _______ | | | | | | | | | | | |
|  |  | | | | | | | | | | | |
| **R5. Your sex:**  *Choose one answer.* | | | | | | | | | | | | |
| 1. | Man | | | | | | | | | | | ⬜ |
| 2. | Woman | | | | | | | | | | | ⬜ |
| **R7. What is your level of education?**  *Choose one answer.* | | | | | | | | | | | | |
| 1. | Primary school or incomplete primary education (up to grades 8-9) | | | | | | | | | | | ⬜ |
| 2. | Basic education (grades 8-9) | | | | | | | | | | | ⬜ |
| 3. | Secondary (completed high school, gymnasium) | | | | | | | | | | | ⬜ |
| 4. | Secondary (completed vocational school, vocational secondary school, technical school) | | | | | | | | | | | ⬜ |
| 5. | Higher (acquired academic higher or professional higher education (bachelor), including completed college, master's degree, doctoral studies) | | | | | | | | | | | ⬜ |
|  |  | | | | | | | | | | |  |
| **R11. What is your work position in your main workplace?**   *The main job is the job that takes up the majority of a person’s their weekly hours. In case a person works multiple part-time jobs, the main job is the one where the salary tax booklet is registered. Attention! If none of the answers correspond to the respondent's position, state it under "Other".* *Choose one answer.* | | | | | | | | | | | | |
| 1. | Higher-level head of the institution, enterprise (executive director, commercial director, chairman of the board) | | | | | | | | | | | ⬜ |
| 2. | Middle-level head (head of the department) | | | | | | | | | | | ⬜ |
| 3. | Higher-level specialist (doctor, teacher, lawyer, architect, senior accountant) | | | | | | | | | | | ⬜ |
| 4. | Specialist (nurse, laboratory technician, technician, inspector, rapporteur, assistant) | | | | | | | | | | | ⬜ |
| 5. | Service and sales worker (secretary, librarian, postal worker, salesman, customer service specialist, hairdresser, police officer, firefighter) | | | | | | | | | | | ⬜ |
| 6. | Skilled worker and craftsman; equipment and machine operator (builder, mechanic, confectioner, seamstress, carpenter) | | | | | | | | | | | ⬜ |
| 7. | Unskilled worker (cleaner, janitor, courier, duty officer, sanitary) | | | | | | | | | | | ⬜ |
| 8. | Other (please specify) | | | | | | | | | | | ⬜ |
